# Ontology-based prediction of cancer driver genes

**DOI:** 10.1038/s41598-019-53454-1

**Published:** 2019-11-22

**Authors:** Sara Althubaiti, Andreas Karwath, Ashraf Dallol, Adeeb Noor, Shadi Salem Alkhayyat, Rolina Alwassia, Katsuhiko Mineta, Takashi Gojobori, Andrew D. Beggs, Paul N. Schofield, Georgios V. Gkoutos, Robert Hoehndorf

**Affiliations:** 1 Computer, Electrical and Mathematical Science and Engineering Division, King Abdullah University of Science and Technology, Thuwal, 23955 Saudi Arabia; 2 Computational Bioscience Research Center, King Abdullah University of Science and Technology, Thuwal, 23955 Saudi Arabia; 3 College of Medical and Dental Sciences, Institute of Cancer and Genomic Sciences, University of Birmingham, B15 2TT Birmingham, United Kingdom; 4 Institute of Translational Medicine, University Hospitals Birmingham, NHS Foundation Trust, B15 2TT Birmingham, United Kingdom; 5 Centre of Excellence in Genomic Medicine Research, King Abdulaziz University, Jeddah, Saudi Arabia; 6 Department of Information Technology, Faculty of Computing and Information Technology, King Abdulaziz University, Jeddah, 80221 Saudi Arabia; 7 Faculty of Medicine, King Abdulaziz University, Jeddah, 21589 Saudi Arabia; 8 Radiation Oncology Unit, King Abdulaziz University Hospital, Jeddah, Saudi Arabia; 9 Biological and Environmental Science and Engineering Division, King Abdullah University of Science and Technology, Thuwal, 23955 Saudi Arabia; 10 Department of Physiology, Development & Neuroscience, University of Cambridge, Downing Street, CB2 3EG Cambridge, United Kingdom; 11 NIHR Experimental Cancer Medicine Centre, B15 2TT Birmingham, UK; 12 NIHR Surgical Reconstruction and Microbiology Research Centre, B15 2TT Birmingham, UK; 13 MRC Health Data Research UK (HDR UK) Midlands, Birmingham, United Kingdom

**Keywords:** Gene ontology, Machine learning, Cancer genetics

## Abstract

Identifying and distinguishing cancer driver genes among thousands of candidate mutations remains a major challenge. Accurate identification of driver genes and driver mutations is critical for advancing cancer research and personalizing treatment based on accurate stratification of patients. Due to inter-tumor genetic heterogeneity many driver mutations within a gene occur at low frequencies, which make it challenging to distinguish them from non-driver mutations. We have developed a novel method for identifying cancer driver genes. Our approach utilizes multiple complementary types of information, specifically cellular phenotypes, cellular locations, functions, and whole body physiological phenotypes as features. We demonstrate that our method can accurately identify known cancer driver genes and distinguish between their role in different types of cancer. In addition to confirming known driver genes, we identify several novel candidate driver genes. We demonstrate the utility of our method by validating its predictions in nasopharyngeal cancer and colorectal cancer using whole exome and whole genome sequencing.

## Introduction

The natural history of cancer is complex^1^ and its genetics highly heterogeneous^2^. Carcinogenesis depends on the accumulation of, and selection for, mutations in genes which are subsequently able to drive, amongst other processes, cell proliferation, immune evasion, genomic instability, and invasiveness. The concept of a cancer driver gene captures the mechanistic requirement for a cell to overcome normal cellular and tissue homeostatic mechanisms and initiate or promote neoplasia and malignancy. The mutations in driver genes are often recessive loss-of-function lesions, exemplified by tumor suppressor genes, but they can also act dominantly as oncogenes. Determining which mutations or genes act as bottlenecks in the generation of cancer is fraught with problems, as cells carrying one or more driver mutations will also carry a large set of co-selected, “passenger”, mutations which constitute most of the normal somatic mutation-load of the expanded cancer cell but which do not directly generate the neoplastic phenotype^3^.

Much effort has gone into developing algorithms to identify driver genes and their mutations, most of which are based on the frequency or pattern of mutations in multiple tumors and their predicted pathogenicity. The goal of identifying cancer drivers may be achieved at the level of gene, protein or pathways, and multiple approaches have been attempted to date^4^. There is no gold standard against which the success of an algorithm can be measured, although the Cancer Gene Census approaches a “gold standard” most closely, with an expert-curated dataset of cancer-associated genes and mutations^5^.

Investigators have increasingly relied on taking a consensus of multiple methods and, where possible, attempted to experimentally verify driver gene status in cellular or whole organism systems^6^. Many thousands of tumors have now been sequenced in very large-scale studies of multiple cancer types, and several hundred genes and mutations have been identified as “drivers” – with varying support from experimental and genetic studies^6^. These methods, however, do not work well for low-intermediate and rare driver genes which may bear up to 20% of driver mutations^7^, and the identification of drivers in specific cancers and sub-types of tumor remains difficult, often because of small numbers of tumors available.

An alternative strategy to sequence-based ratiometric type mutation frequency based approaches is to identify a “fingerprint” for cancer driver genes from a range of biological and molecular data and to use this as part of a classifier that can filter sequence information. For example, it is possible to utilize gene annotations and biological properties of known driver genes in a machine learning approach and identify novel driver gene candidates^8^. Here, we report a novel method in which we use a combination of direct functional evidence, obtained through cell growth assays after introducing a specific mutation into cells, and related functional characteristics available from, for example, experiments using model organisms, to build a classifier that determines whether a gene is likely to become a cancer driver gene.

Public databases contain large volumes of information that relates genes or variants to phenotypes (either on the cellular or whole body organism level), the specific biological processes and molecular functions in which they can be involved, or the cellular locations at which a gene product is active. Phenotypes are systematically collected in the context of genotype–phenotype relations, both from human clinical information^9^, from model organism experiments^10^, and for cell models in cellular phenotype databases^11^. Information about gene functions is collected in databases such as Uniprot^12^ as well as several model organism databases.

In these databases, ontologies are used for characterizing phenotypes, gene functions and cellular locations. Over the past two decades a tightly integrated system of ontologies has been developed that interlinks knowledge about basic biological phenomena through the use of logical axioms^13^. Exploring the information in this system of ontologies can enable novel types of analysis^14^ and the background knowledge in the ontologies has the potential to significantly improve biomedical data analysis^15^.

We have developed a method that uses biological background knowledge about the relations between genes or variants and their phenotypes, either on the cellular or whole organism level, as well as gene functions and cellular locations, to predict driver genes and mutations. Our approach relies on neuro-symbolic deep learning to systematically encode background knowledge about basic biological processes and phenomena. Specifically, we generate “embeddings” for gene functions and gene–phenotypes associations and use a deep artificial neural network trained on known driver genes – and the knowledge about how they relate to functions and phenotypes – to discover new cancer drivers. We demonstrate that our method can predict novel driver genes by analyzing two cohorts of different cancers, and we demonstrate that the predicted driver genes have a significantly higher somatic mutation frequency, are significantly more likely to be functionally related to known drivers, and have a significantly higher rate of pathogenic somatic variants. We make our method and prediction results freely available from https://github.com/bio-ontology-research-group/predCAN.

## Results

### Representation learning

Our aim is to utilize information about the functions and phenotypes associated with genes to identify cancer driver genes and, subsequently, driver mutations. This information is represented using biomedical ontologies, and these ontologies also contain a substantial amount of background information about the relations between biological functions, processes, and phenotypes in the form of logical axioms and natural language definitions^14^. The information in ontologies is utilized by human experts to understand and interpret the implications of an association with a class in an ontology, and a comprehensive interpretation of these associations relies on comprehension and utilization of biological background knowledge.

We use three types of information associated with genes: cellular phenotypes observed in large-scale microscopy studies and recorded using the Cellular Microscopy Phenotype Ontology (CMPO)^16^; gene functions and cellular locations recorded by Uniprot^12^ and encoded using the Gene Ontology (GO)^17^; and phenotypes of knockout mouse models provided by the Mouse Genome Informatics (MGI) database^10^ and encoded using the Mammalian Phenotype Ontology (MP)^18^.

**Figure 1:**
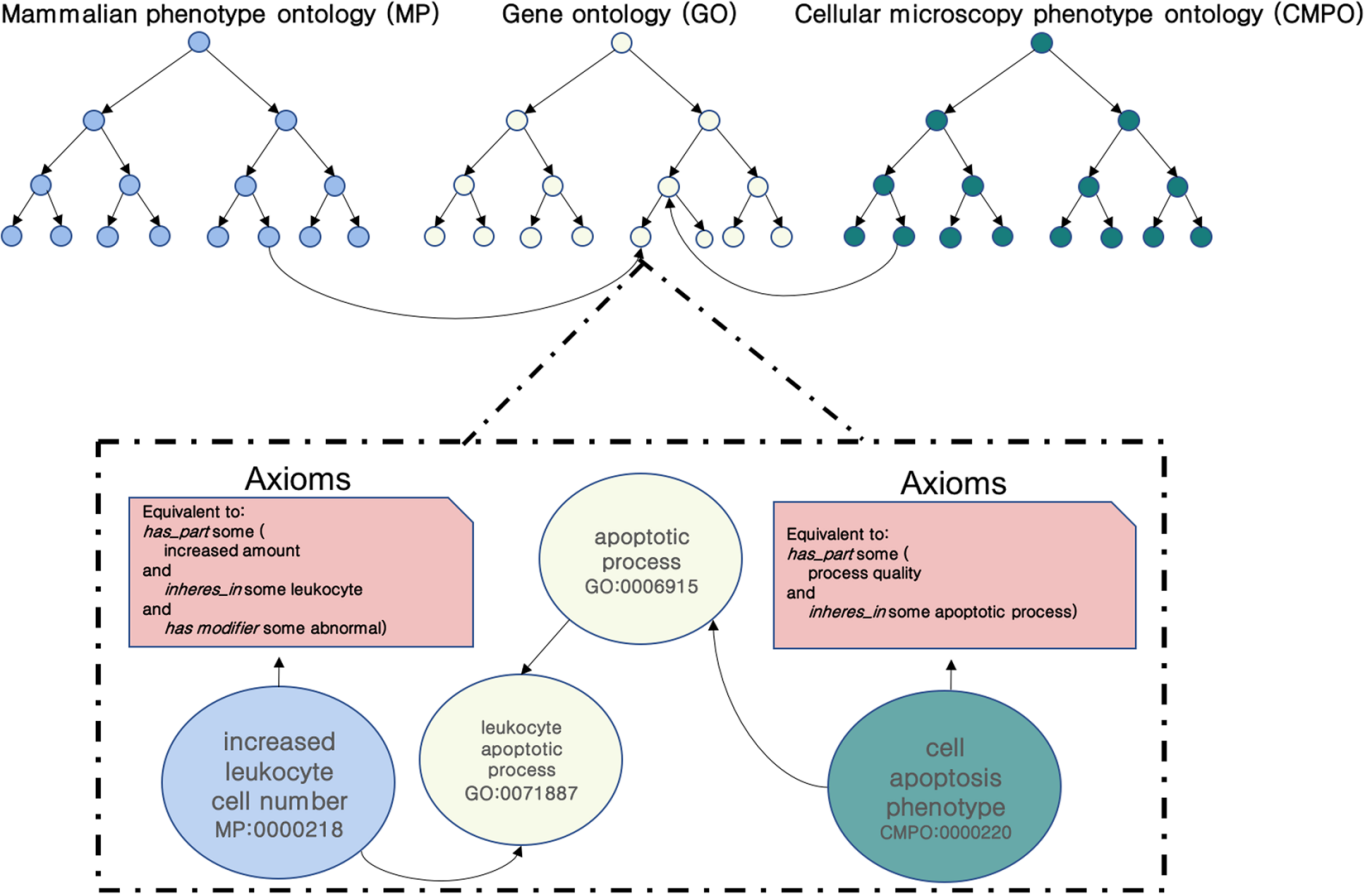
Interlinked knowledge between ontologies.

Each of these ontologies contains logical axioms that define and restrict the classes and provide background knowledge about the domain of functions, cellular phenotypes, or physiological phenotypes^18^. Figure 1 shows an example of how the ontologies overlap in their content and how classes in phenotype ontologies are defined or constrained using classes from other ontologies.

We have developed a method that combines the associations of genes and ontology classes with the background information in ontologies (both the formal logical content and the informal natural language components) into single feature vectors that represent each gene in our dataset. For this purpose, we utilize neuro-symbolic feature learning on ontologies in which machine learning models are combined with deductive inference on formally represented knowledge^19^; as a result, we obtain an embedding – a function from an ontology and its associated entities into an *n*-dimensional vector space – which generates feature vectors that encode for known associations of genes and their functions or phenotypes as well as the ontologies’ background knowledge.

**Figure 2:**
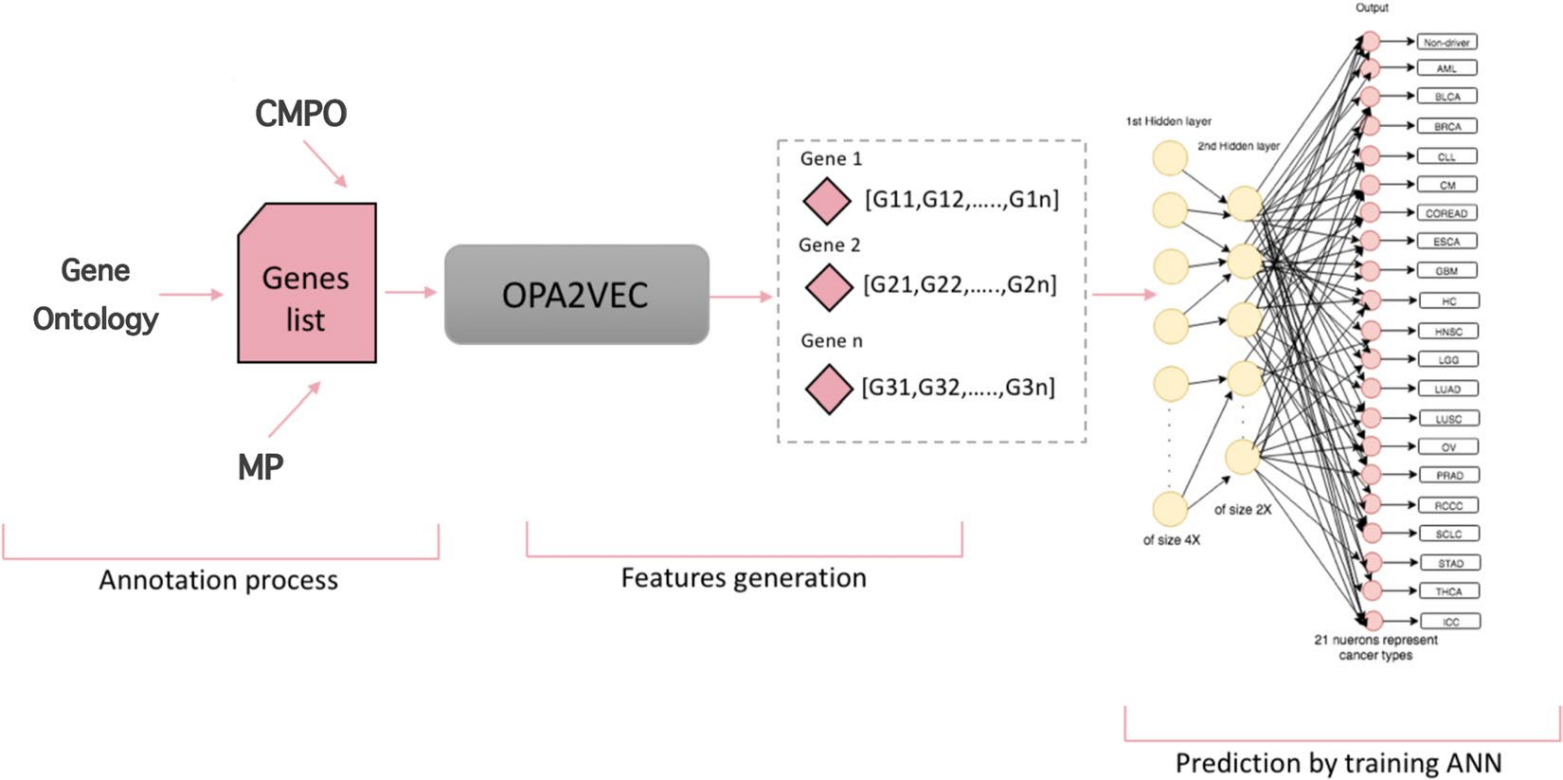
Overall workflow. Starting with annotation the genes from CPD with CMPO, GO and MP; and generates vectors with OPA2Vec. The prediction process done by training ANN in order to which cancer type each gene belong to discover new driver genes.

Because different genes are covered differently in the databases we use, we generate five different representations for each gene. The first three representations are based on annotating the genes using the ontologies that we used one at a time, combining the ontology-based annotations and the ontology axioms within a single representation so that the background knowledge within the ontologies becomes accessible. However, we can only generate the feature vectors if there are ontology-based annotations for a gene and therefore we obtain a different number of feature vectors when utilizing different ontologies. To determine if we can improve our predictions we combine the embeddings generated from each gene if all three features are available and evaluate the performance on the intersection of genes for which features in all three ontologies can be generated. Finally, we determine if it is possible to utilize the ontology axioms. For this purpose, we merge the three ontologies (CMPO, GO, MP) into a single ontology model and generate the embeddings for each gene which is annotated to information in at least one of the three ontologies we use. We use the generated feature vectors as input to a deep neural network that we train to predict cancer driver genes and distinguish between 20 cancer types for which a gene can be considered a driver. Figure 2 illustrates our workflow.

### Biological features and background knowledge predict cancer drivers

We first test how well each type of feature predicts cancer driver genes separately. For this purpose, we construct a machine-learning model to classify genes into driver genes for particular cancer types. We train this model using the known driver genes and non-driver genes available from the IntOGen^20^ database. We distinguish between 20 different types of cancer (see Supplementary Table 1) taken from the IntOGen database^20^. The machine learning model we use is illustrated in Supplementary Fig. 1. We evaluate the results using 10-fold cross-validation and performance results are summarized in Table 1.

**Table 1:** ANN performance in identification driver/non-driver genes of different cancer types.

Experiments	F-score	AUC	Number of annotated genes
CMPO	78.95%	72.96%	13,116
GO	84.23%	78.81%	17,591
MP	85.39%	79.51%	7,884
CMPO + GO + MP (Intersection)	91.20%	89.73%	7,884
CMPO + GO + MP (Union)	**92**.**57%**	**94**.**28%**	20,352

We first determine the performance using each ontology-based feature separately (using each ontology individually as background knowledge), and then determine the performance only on genes for which we have all three ontology-based features (Intersection); in the “Intersection” case, embedding vectors are concatenated. Finally, we determine the performance on genes for which we have at least one feature (Union); in the latter case, ontologies are merged.

**Figure 3:**
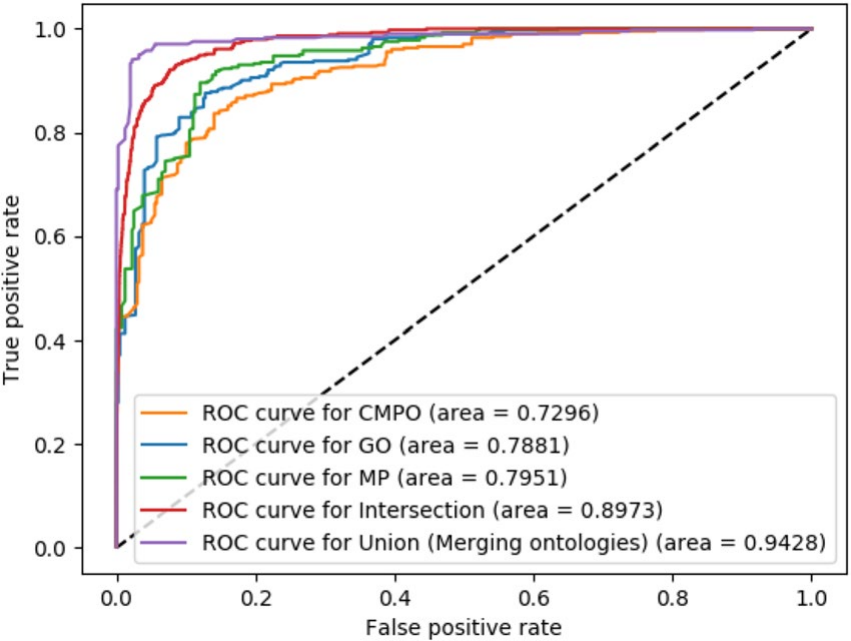
ROC curves and AUC values for the prediction of driver genes based on different ontology associations.

**Figure 4:**
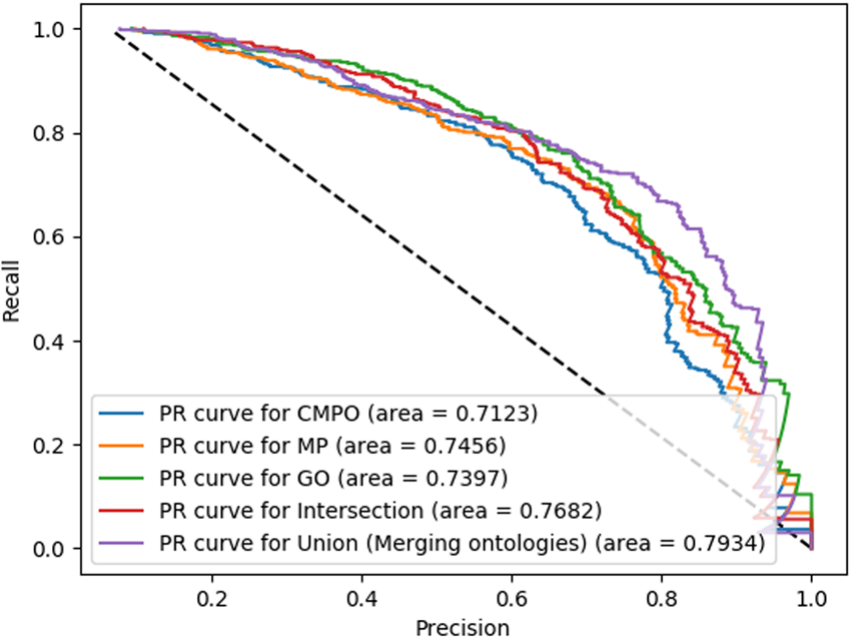
Precision–recall curves for the prediction of driver genes based on different ontology associations.

While the individual features can be used to predict driver genes in our experiment, we hypothesize that the different ontology-based features contain complementary information. Therefore, we merge the ontologies in a single ontology by combining the axioms from CMPO, GO, and MP The ontologies contain background knowledge about their respective domains, and combining the ontologies allows us to combine the ontology-based annotations of each gene as well. When merging the ontologies and annotations we achieve a significant improvement of prediction results compared to predicting based on individual features (see Table 1, Fig. 3 for ROC curves and Fig. 4 for the precision-recall curves). As our method can accurately predict cancer driver genes, we apply our model to all human genes for which we have ontology-based annotations and predict 112 novel candidate driver genes for 20 different cancer types (Supplementary Tables 2 and 3).

### Predicted cancer driver genes are hypermutated in cancer exomes

Driver genes will generally accumulate more mutations than other genes in a tumor^21^. We use the complete list of genes in the IntOGen database and separate them into three disjoint groups: genes listed as confirmed cancer drivers, genes predicted as cancer drivers for one or more tumor types by our method but not known to be a driver gene, and genes not known or predicted as cancer driver genes. We use the recorded somatic mutations in the IntoGen database to determine the somatic mutation frequency for each human gene (i.e., the number of mutations divided by the length of the gene). We find that candidate predicted driver genes by our method have a significantly higher somatic mutation frequency than non-driver genes ($p=0.560\times {10}^{-12}$, one-tailed t-test).

We also evaluate our predictions on a set of 26 tumor samples for nasopharyngeal cancer obtained from patients at King Abdulaziz University Hospital in Jeddah, Saudi Arabia, for which we have performed whole exome sequencing. Nasopharyngeal cancer is a type of squamous cell carcinoma of the head and neck for which we identify seven novel candidate driver genes (see Supplementary Table 3). In this set of samples, the predicted driver genes have a significantly higher somatic mutation frequency compared to non-driver genes ($p=0.115\times {10}^{-5}$, one-tailed t-test).

Moreover, we applied the same analysis to 114 tumor/normal samples for colorectal adenocarcinoma obtained from the University of Birmingham Hospital in UK. We identified 12 candidate driver genes for colorectal adenocarcinoma. Among the 114 samples, the predicted driver genes have a significantly higher somatic mutation frequency compared to non-driver genes ($p=0.204\times {10}^{-3}$, one-tailed t-test).

### Predicted cancer driver genes are functionally related to known cancer drivers

Cancer drivers are known to form functional modules on interaction networks^22^. We use the STRING interaction network^23^ to determine whether the genes we predicted are functionally related to known driver genes. STRING contains several types of interaction between genes and proteins, including physical interactions, genetic interactions, co-location, and co-expression.

We find that 24 out of the 112 candidate driver genes have a direct interaction with a known driver gene within their respective cancer type. In a random distribution (see Methods), on average six genes are connected to a known driver gene, demonstrating that our predicted drivers are significantly more likely to be functionally related to a known driver gene ($p=0.731\times {10}^{-7}$, one-tailed t-test).

### Predicted driver genes are enriched for pathogenic variants

Our method can also be used to detect driver variants in tumor samples. We use seven different methods for predicting deleteriousness of variants which have previously been used in large-scale evaluations of driver variants^6^. We compare the pathogenicity scores of variants in candidate driver genes to known and non-driver genes in nasopharyngeal carcinoma exomes. Variants in the seven predicted driver genes for head and neck squamous cell carcinoma generally are scored more pathogenic than variants in non-driver genes by most prediction tools (SIFT: $p=0.324\times {10}^{-5}$, PolyPhen-2: $p=0.098$, MutationAssessor: $p=0.378\times {10}^{-6}$, MutationTaster: $p=0.372\times {10}^{-10}$, CADD: $p=0.818$, VEST3: $p=0.169\times {10}^{-12}$ and FATHMM: $p=0.912\times {10}^{-14}$; Mann-Whitney U test).

We apply the same test on the cohort of colorectal adenocarcinoma samples. The set of variants in the predicted driver genes for colorectal adenocarcinoma (see Supplementary Table 4) are scored as significantly more pathogenic than variants in non-driver genes by most pathogenicity prediction methods (SIFT: $p=0.631\times {10}^{-6}$, PolyPhen-2: $p=0.725\times {10}^{-8}$, MutationAssessor: $p=0.405\times {10}^{-2}$, MutationTaster: $p=0.917$, CADD: $p=0.811$, VEST3: $p=0.040$ and FATHMM: $p=0.648\times {10}^{-8}$; Mann-Whitney U test).

Most of the prediction methods predict variants in candidate driver genes as significantly more pathogenic than in non-driver genes. However, not all evaluation methods show significant results for both cohorts. Notably, CADD does not show a significant enrichment for pathogenic variants in either dataset, PolyPhen-2 does not show this enrichment in predicted nasopharyngeal driver genes, and MutationTaster does not show this enrichment in predicted colorectal adenocarcinoma driver genes. A possible explanation may be that CADD has been trained to determine deleterious effects in germline variants^24^, and PolyPhen-2 as well as MutationTaster were trained to detect Mendelian disease variants^25,26^, while we apply these methods to somatic mutations. MutationAssessor and FATHMM, on the other hand, were specifically designed to evaluate somatic mutations^27,28^.

We can also use the pathogenicity prediction methods to suggest candidate driver mutations in the cohorts as well as individual patients. Supplementary Tables 4 and 5 list all rare variants in predicted driver genes in the predicted driver genes for nasopharyngeal carcinoma and colorectal cancer. For example, *TGM3* has previously been studied as candidate driver in carcinomas of the head and neck^29^, and we identify rs200294064 in *TGM3* as pathogenic candidate driver mutation.

## Discussion

Our method can predict cancer driver genes using only public background knowledge about gene functions, cellular and organism phenotypes. The key novelty of our algorithm is the ability to encode biological background knowledge in ontologies^15^, and therefore exploit inter-ontology links in the form of axioms; the predictive performance of our method is best when combining different – yet related – ontologies. While many of the axioms that relate classes in different ontologies have been created to support ontology development and maintenance, we show that ontologies in the biomedical domain now form a comprehensive web of biological background knowledge that can – when exploited through appropriate learning algorithms – significantly improve the interpretation and analysis of data. A crucial role for this type of connection between different ontologies is the Relation Ontology^30^ which is central to ontologies that make up the OBO Foundry^13^.

Through the application of our method we identify novel driver genes by systematically analyzing public knowledge on multiple levels of granularity. One of the key novelties in our approach is the use of phenotype data – both on the cellular and whole organism levels of granularity – to characterize cancer driver genes. We use sequencing data – both public and patient-derived – as validation. This strategy is the opposite of most computational and experimental approaches in which molecular data is used as feature and additional functional evidence collected after predictions^6,31^. Our predictions determine the potential of a gene to play a role as a cancer driver in particular tumor types, and is independent of specific information regarding stage or gender.

Our approach can identify consensus cancer driver genes previously identified and not used in our training data; For example, 8 out of the 112 genes we predict have been listed in the current COSMIC database of confirmed consensus cancer driver genes^32^ (see Supplementary Table 6). Moreover, we found 12 out of the 26 rare variants within the predicted driver genes for both nasopharyngeal and colorectal cohorts mentioned in International Cancer Genome Consortium (ICGC) data^33^ (see Supplementary Table 7). We further compare our prediction results to a dataset in which 7,470 human genes have been modified through CRISPR/Cas9 in 324 cell lines for 19 different tissues to determine driver genes^34^. We find that 47 out of our 112 candidate genes overlap regardless of their cancer type (${\rm{p}}=0.22\times {10}^{-17}$), and 16 out of the 112 candidate genes overlapped within the same cancer type.

## Conclusions

Cancer driver genes are commonly identified using computational methods applied to tumor-derived sequence data, or experimentally based on introducing targeted mutations in cell models. We developed a novel approach that computationally predicts driver genes based on known functions and phenotypes associated with genes as well as biological background knowledge contained in ontologies, and we evaluate the predictions using sequencing data from two cohorts of tumor patients. Our method relies on deep learning over integrated ontologies and biological knowledge bases, and highlights the importance of using structured and formalized resources as background knowledge in machine learning models.

## Materials and Methods

### Data sources and ontologies

We performed all our experiments on a set of driver/non-driver genes from the Mutational Cancer Drivers Database (intOGen)^20^ and Cellular Phenotype Database (CPD)^11^ downloaded on 18 Feb 2018. They contain a total of 60,279 genes: 24,475 of them are human protein-coding genes and the remaining 35,804 represent different types of RNA molecules and pseudogenes. Some of the genes are identified as being driver genes in one of the 28 cancer types in intOGen.

Furthermore, we used cellular phenotype annotations of genes provided by the Cellular Phenotype Database (CPD)^11^, downloaded on 18 Feb 2018, and we used function annotations provided by the Gene Ontology (GO) website^17^ downloaded on 14 Jul 2018. We further used phenotype annotations observed in mutant mouse models, downloaded from the Mouse Genome Informatics (MGI) database^10^ on 14 Jul 2018.

We mapped all gene identifiers to Entrez gene identifiers and used the mapping file between Ensembl gene identifiers and Entrez genes provided by the Cellular Phenotype Database (CPD)^11^; we converted UniProt identifiers of proteins in the GO annotations to Entrez gene identifiers using the UniProt mapping tool provided on the UniProt website^12^; and for assigning phenotypes to genes we use mouse orthologs of human genes and assign the phenotypes associated with loss-of-function mutations in mice in the MGI database^10^ to the human genes.

We started with 20,352 human genes of which 13,116 are annotated using the Cellular Microscopy Phenotype Ontology (CMPO)^16^, 17,591 are annotated using the Gene ontology (GO)^17^, and 7,884 are annotated with the Mammalian Phenotype Ontology (MP)^18^.

### Generation of ontology annotation-based features

We used the OPA2Vec approach^19^ to generate “embeddings” representing genes based on the different ontologies and the annotations of the genes with the ontologies. An ontology embedding is a function that projects entities in an ontology or annotated with an ontology into an *n*-dimensional real-valued space while preserving some of the ontology’s structure.

For generating the ontology embeddings using OPA2Vec, we evaluated different embedding sizes and minimum count parameters while fixing a window size of 5; the majority of axioms within the ontology used by OPA2Vec workflow contain 3 words (i.e., simple subclass axioms) and almost never exceed 10 words, for which a window size of 5 suffices. Among our experiments (see Supplementary Table 8), the optimal combination of parameters are and embedding size 100, minimum count 5, and using the default skip-gram model. We fixed these parameters throughout all our experiments.

### Supervised training

We investigated the performance of a machine learning based classification algorithm in identifying new driver genes. In our experiments, we used known driver genes within 20 different cancer types as the positive set, and randomly selected an equal number of the non-driver genes as the negative set.

For the training and testing, we performed stratified 10-fold cross-validation. We performed a limited grid search for optimal sets of hyperparameters of the neural network using the Hyperas system^35^. We used a Rectified Linear Unit as an activation function^36^ for the hidden layers and a sigmoid function as the activation function for the output layer; we used cross entropy as loss function in training, and Rmsprop^37^ to optimize the neural networks parameters in training.

### Sample preparation and sequencing

For nasopharyngeal cancer samples: 26 patients with nasopharyngeal cancer received treatment at King Abdulaziz University Hospital, Jeddah. 16/26 were male and 10/26 female. Patients has tumor that ranges from T1-4 and nodal involvement from N0-3 (22/26 patients). Four patients had metastatic disease on radiological assessment (4/26). 20 out of 26 patients received combined treatment with curative intension of which 8/26 patients received concurrent chemoradiation and 12/26 patients received induction chemotherapy followed by concurrent chemoradiation. Two patients could not receive any active treatment and were offered only palliative measures. Four patients had metastatic disease from which 2 received palliative chemotherapy and the other 2 patients received induction chemotherapy followed by chemoradiation for aggressive palliation as they had good response to initial chemotherapy.

Tissue samples were taken from nasopharyngeal cancer lesions and embedded in formalin at King Abdulaziz University Hospital in Jeddah, KSA. DNA was extracted from the tissue using Qiagen QIAamp FFPE Tissue DNA extraction kit (56404) following the manufacturer’s instruction. To capture the exomes and to prepare the sequencing libraries, a TruSeq exome kit (Illumina) was used. The indexed libraries were pooled (7 samples per lane), and 7 lanes in total were used for paired-end sequencing (150 bp) on a HiSeq. 4000 (Illumina). The average sequencing depth is 237x.

For colorectal carcinoma samples: In the patients studied, all had primary colorectal cancer, 34/54 were male and 20/54 were female. Two patients with rectal cancer underwent neoadjuvant chemoradiotherapy and one underwent neoadjuvant short course radiotherapy before excision of the primary tumour. The pathological stage of the resected tumours varied from between T2N0 to T4N2. Five patients presented with metastatic disease and 18 patients had “high risk” disease consisting of any of poor differentiation (4 patients), extra-mural vascular invasion (18 patients), or threatened circumferential resection margin (2 patients). The operation types were abdomino-perineal excision of rectum (1 patient), anterior resection of rectum (15/54), left hemicolectomy (5/54), panproctocolectomy (1 patient), right hemicolectomy (14/54), sigmoid colectomy (4/54) and subtotal colectomy (2/54).

Colorectal cancer tissue and paired germline samples were obtained at the time of surgery and snap frozen in liquid nitrogen until required. For whole genome sequencing, approximately 1–3 *μ*g of DNA was sheared and prepared using an Illumina TruSeq PCR-free library preparation kit. Samples were indexed and each sample was pooled across 4 lanes of an Illumina HiSeq 4000 using v4 sequencing chemistry, generating approximately 150 Gb of sequencing data per sample using a 125 bp PE sequencing strategy at an average depth of 53.7x.

### Generating and annotating variant calls

For nasopharyngeal cancer samples we used GATK with Mutect2^38^ for sample preparation and generating somatic short variant calling files following tumor-only mode. For colorectal carcinoma, Strelka^39^ was used with tumor/normal pairs to detect somatic mutations. We filter all variants that do not lie within a coding region in both cohorts.

We annotate the resulting VCF files with Annovar^40^ to extract variant-related information such as the gene(s) in which a variant lies and the start and end position of the gene, and the different pathogenicity scores.

### Detection of network modules

To determine a neutral distribution of the number of connections between cancer driver genes within STRING empirically, we repeatedly sample 112 random nodes from the STRING interaction network^23^ and calculate the number of genes which are connected to known cancer driver genes. We repeat this process 10,000 times^41^.

### Ethical considerations and consent

This work has been reviewed and approved by the Research Ethics Committee of King Abdulaziz University on 29 March 2017 under reference number 116-17, and the Institutional Bioethics Committee at King Abdullah University of Science and Technology on 15 May 2017 under reference number 17IBEC07-Hoehndorf. Ethical approval for work on colorectal cancer samples was obtained via the University of Birmingham Human Biomaterials Resource Centre Biobanking ethics (Ref 09/H1010/75). Informed consent was obtained from all subjects included in this study. All methods were carried out in accordance with the guidelines and regulations laid out by the institutional bioethics committees, the Declaration of Helsinki, and applicable laws and regulations governing research involving human subjects.

## Supplementary information

Supplementary Information

## Data Availability

All data except human genomic data is freely available from https://github.com/bio-ontology-research-group/predCAN. Genomic data for nasopharyngeal and colorectal cancer samples cannot be shared publicly due to ethical and legal restrictions. Requests for access can be sent to the University of Birmingham Human Biomaterials Resource Centre Biobanking and the Research Ethics Committee of King Abdulaziz University in Jeddah which will decide on access for researchers who meet the criteria for access to confidential data.
