# Supplementary material for: Ontology-based prediction of cancer driver genes
Source: Sci Rep. 2019 Nov 22;9:17405. doi: 10.1038/s41598-019-53454-1 (PMC6874647; doi:10.1038/s41598-019-53454-1)
Supplement: Supplementary file 1 — Supplementary Information [file 41598_2019_53454_MOESM1_ESM.pdf]

Ontology-based prediction of cancer driver genes

Sara Althubaiti[1,2], Andreas Karwath[3,4], Ashraf Dallol[5], Adeeb Noor[6], Shadi Salem Al-Khayyat[7], Rolina Alwassia[8], Katsuhiko Mineta[1,2], Takashi Gojobori[2,9], Andrew D. Beggs[3], Paul N. Schofield[10], Georgios V. Gkoutos[3,4,11,12,13], and Robert Hoehndorf[1,2,*]

[1]Computer, Electrical and Mathematical Science and Engineering Division, King Abdullah University of Science and Technology, Thuwal 23955, Saudi Arabia
[2]Computational Bioscience Research Center, King Abdullah University of Science and Technology, Thuwal 23955, Saudi Arabia
[3]College of Medical and Dental Sciences, Institute of Cancer and Genomic Sciences, University of Birmingham, B15 2TT, Birmingham, United Kingdom
[4]Institute of Translational Medicine, University Hospitals Birmingham, NHS Foundation Trust, B15 2TT, Birmingham, United Kingdom
[5]Centre of Excellence in Genomic Medicine Research, King Abdulaziz University, Jeddah, Saudi Arabia
[6]Department of Information Technology, Faculty of Computing and Information Technology, King Abdulaziz University, Jeddah 80221, Saudi Arabia
[7]Faculty of Medicine, King Abdulaziz University, Jeddah 21589, Saudi Arabia
[8]Radiation Oncology Unit, King Abdulaziz University Hospital, Jeddah, Saudi Arabia
[9]Biological and Environmental Science and Engineering Division, King Abdullah University of Science and Technology, Thuwal 23955, Saudi Arabia
[10]Department of Physiology, Development & Neuroscience, University of Cambridge, Downing Street, CB2 3EG, Cambridge, United Kingdom
[11]NIHR Experimental Cancer Medicine Centre, B15 2TT, Birmingham, UK
[12]NIHR Surgical Reconstruction and Microbiology Research Centre, B15 2TT, Birmingham, UK
[13]MRC Health Data Research UK (HDR UK), United Kingdom
[*]robert.hoehndorf@kaust.edu.sa

Table 1: 20 different cancer types have been used

Corresponding Class	Cancer Type	Number of genes
0	Not identified	19,893
1	Acute myeloid leukemia (AML)	32
2	Bladder carcinoma (BLCA)	156
3	Breast carcinoma (BRCA)	184
4	Chronic lymphocytic leukemia (CLL)	38
5	Cutaneous melanoma (CM)	250
6	Colorectal adenocarcinoma (COREAD)	95
7	Esophageal carcinoma (ESCA)	98
8	Glioblastoma multiforme (GBM)	75
9	Hepatocarcinoma (HC)	30
10	Head and neck squamous cell carcinoma (HNSC)	167
11	Lower grade glioma (LGG)	50
12	Lung adenocarcinoma (LUAD)	181
13	Lung squamous cell carcinoma (LUSC)	147
14	Ovarian Carcinoma (OV)	83
15	Prostate adenocarcinoma (PRAD)	88
16	Renal clear cell carcinoma (RCCC)	105
17	Small cell lung carcinoma (SCLC)	61
18	Stomach adenocarcinoma (STAD)	175
19	Thyroid carcinoma (THCA)	32
20	Uterine corpus endometrioid carcinoma (ICC)	149

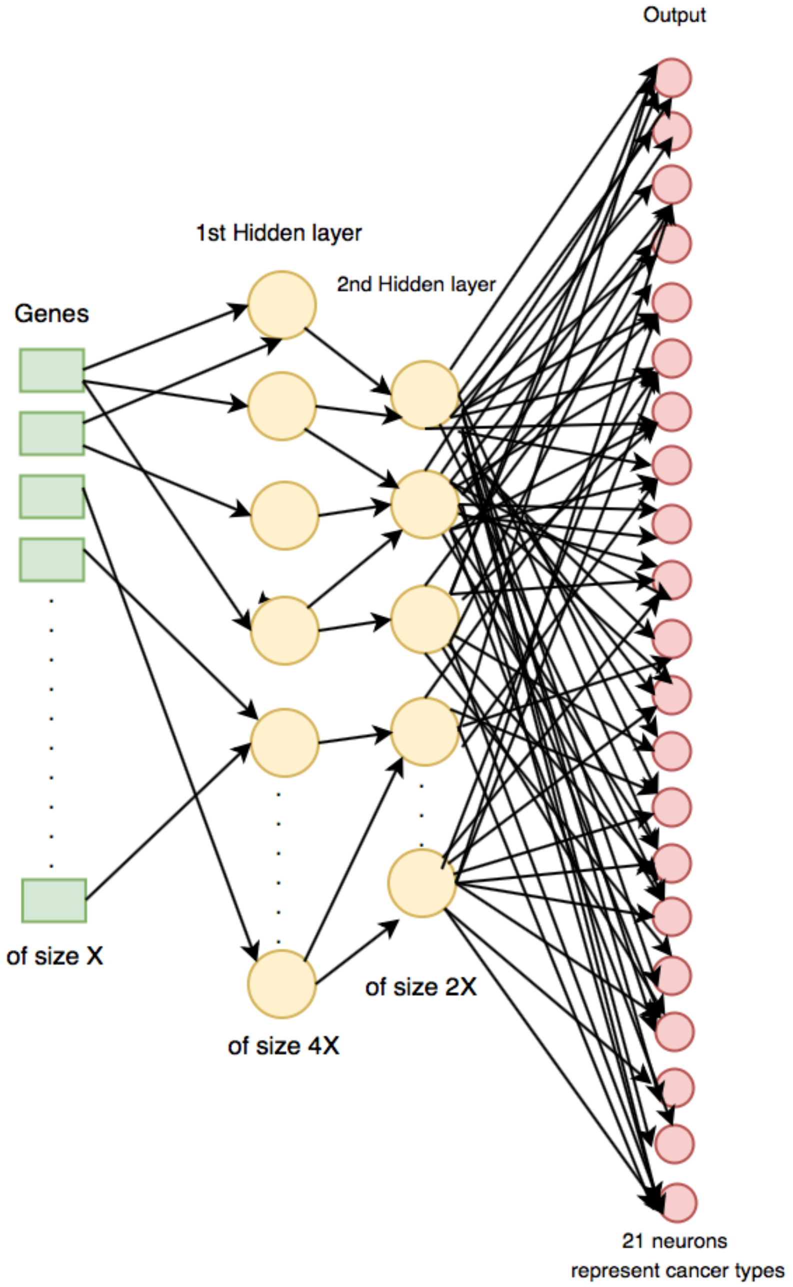

Figure 1: Neural network architecture consisting of an input layer of the embedding size x for one gene, and two hidden layers of size $4x$ and $2x$, and the output layer consisting of 21 neurons for each of the 20 cancer types plus one class to classify a gene as non-driver.

Table 2: 20 different cancer types have been used

Corresponding Class	Cancer Type	Number of genes
0	Not identified	19,893
1	Acute myeloid leukemia (AML)	32
2	Bladder carcinoma (BLCA)	156
3	Breast carcinoma (BRCA)	184
4	Chronic lymphocytic leukemia (CLL)	38
5	Cutaneous melanoma (CM)	250
6	Colorectal adenocarcinoma (COREAD)	95
7	Esophageal carcinoma (ESCA)	98
8	Glioblastoma multiforme (GBM)	75
9	Hepatocarcinoma (HC)	30
10	Head and neck squamous cell carcinoma (HNSC)	167
11	Lower grade glioma (LGG)	50
12	Lung adenocarcinoma (LUAD)	181
13	Lung squamous cell carcinoma (LUSC)	147
14	Ovarian Carcinoma (OV)	83
15	Prostate adenocarcinoma (PRAD)	88
16	Renal clear cell carcinoma (RCCC)	105
17	Small cell lung carcinoma (SCLC)	61
18	Stomach adenocarcinoma (STAD)	175
19	Thyroid carcinoma (THCA)	32
20	Uterine corpus endometrioid carcinoma (ICC)	149

Table 3: Number of predicted driver genes within each cancer type

Cancer Type	Number of predicted genes
AML	2
BLCA	4
BRCA	14
CLL	2
CM	11
COREAD	12
ESCA	3
GBM	4
HC	5
HNSC	7
LGG	1
LUAD	9
LUSC	5
OV	3
PRAD	3
RCCC	7
SCLC	4
STAD	5
THCA	7
ICC	11

Table 4: The list of the predicted driver-genes for each cancer type.

Cancer type	Gene name	Number of mutations	Gene length	Mutation_Frequency
COREAD	MAPK1	33	98652	2989.454545
HNSC	RRAGD	18	47661	2647.83333
CM/LUSC	PRKACB	32	40800	1275
COREAD	STIL	61	64009	1049.327869
UCEC	PKHD1	358	366777	1024.51676
UCEC	ALDH1A1	53	52656	993.509434
BLCA	SLC15A1	74	68875	930.7432432
BRCA	TCF15	7	6274	896.2857143
CM	RBP1	15	13425	895
CM	SCD	20	17817	890.85
HC	PEBP1	11	9705	882.2727273
LUSC	MAPK6	55	47052	855.4909091
UCEC	SLC9A2	110	91817	834.7
LUAD	PLOD1	52	40876	786.0769231
STAD	ETNK1	28	19274	688.357143
HNSC	TGM3	71	45113	635.3943662
SCLC	SLC12A3	82	50644	617.6097561
LGG	SULT2B1	39	23692	607.4871795
GBM/CM	SULT1E1	33	18941	573.969697
BRCA	PRPF19	30	16042	534.7333333
HC	S100A11	9	4530	503.3333333
GBM	PEX5	42	21122	502.9047619
CM	TAL1	32	15426	482.0625
CM	SUV39H1	26	12283	472.4230769
LUAD	PDK4	28	13117	468.4642857
HNSC	TFAP2B	63	28888	458.5396825
STAD	PGM3	60	27164	452.7333333
HNSC	OSGIN1	29	13111	452.103448
HC	SNRPB	21	9219	439
UCEC	CHST6	50	21905	438.1
COREAD	CERCAM	39	16872	432.615385
BRCA	SCNN1G	79	34161	432.4177215
AML	RBM3	16	6813	425.8125
COREAD	SLC28A2	57	23705	415.877193
RCCC	ALG12	37	15253	412.243243
UCEC	PITX3	28	11286	403.0714286
COREAD	PPM1G	74	28485	384.9324324
CM/PRAD	PFKM	62	23758	383.1935484
LUSC/UCEC	RIT1	35	13108	374.5142857
STAD	RUNX1T1	168	62714	373.297619
COREAD	OBSCN	412	153121	371.652913
COREAD	SFTPB	31	11425	368.5483871
BRCA	POU3F1	8	2928	366
BRCA	CXCL12	23	8036	349.3913043
SCLC	COX17	24	7872	328
COREAD	ZKSCAN2	66	21534	326.272727
UCEC	SOX11	27	8719	322.9259259
THCA	PIP	24	7661	319.2083333
PRAD/STAD/LUAD	PPP1R10	57	16907	296.6140351
STAD	SRY	3	887	295.6666667
OV	POLR2A	106	30238	285.2641509
COREAD	SNRPD2	16	4539	283.6875
RCCC	XCL1	20	5605	280.25
LUSC	SLC9A5	85	23240	273.411765
LUAD	PIM1	21	5283	251.5714286
BLCA/RCCC	SFRP2	34	8487	249.6176471
CM	STC1	53	12887	243.1509434
CM	PYCR1	20	4854	242.7
LUAD	ITPKA	40	9702	242.55
OV	POU3F3	19	4506	237.1578947
STAD	SRMS	32	7581	236.90625
HNSC	TNKS1BP1	110	25311	230.1
THCA	PIGR	78	17945	230.0641026
BRCA	SLC52A3	37	8505	229.864865
BLCA	PLXNB1	112	25612	228.6785714
BRCA	HAP1	54	12008	222.3703704
COREAD	BRD8	101	21786	215.70297
ESCA/SCLC	CCL3	9	1904	211.5555556
HC	ANG	26	5414	208.2307692
BRCA	PROP1	20	4008	200.4
HC	PGLYRP2	56	10859	193.9107143
THCA	NOLC1	62	11696	188.6451613
CM	LEFTY2	26	4895	188.2692308
CLL	PTMS	25	4636	185.44
HNSC	CCNA1	62	10611	171.1451613
LUAD	SIX3	26	4180	160.7692308
CM	SRF	59	9117	154.5254237
COREAD	FES	70	10731	153.3
CLL	MAP2K7	73	10704	146.630137
RCCC	GRB7	51	7319	143.5098039
AML	SPTA1	543	76010	139.9815838
BRCA	CCL1	21	2906	138.3809524
THCA	RXRG	64	8428	131.6875
PA	FCER1G	32	3952	123.5
UCEC	PCOLCE	51	5917	116.019608
PRAD	MBD1	94	10733	114.1808511
BRCA/HNSC	TAPBP	104	10743	103.2980769
LUAD	KRT18	38	3843	101.1315789
UCEC	MYH4	294	26269	89.35034014
LUSC	SSTR5	30	2674	89.13333333
RCCC	SPHK1	34	2653	78.02941176
GBM	NES	113	8634	76.40707965
ESCA	SSTR1	67	5065	75.59701493
RCCC	PBX2	74	5451	73.66216216
LUAD	PLP2	47	3285	69.89361702
ESCA	RAG1	180	11748	65.26666667
COREAD	S100A8	15	945	63
UCEC	PNMT	34	2023	59.5
RCCC	FTL	29	1571	54.17241379
SCLC	GLMP	57	3003	52.68421053
THCA	IFNA8	20	1039	51.95
BRCA	SLC1A3	58	2943	50.74137931
LUAD	SOX15	40	1991	49.775
THCA	EPHA10	100	4880	48.8
UCEC	PTPRCAP	53	2173	41
BRCA	DAXX	109	4458	40.89908257
OV	PRM1	13	500	38.46153846
BRCA	FKBPL	45	1584	35.2
THCA	SOD1	332	9309	28.03915663
GBM	SPEG	224	6051	27.01339286
BLCA	PGK2	96	1690	17.60416667
BRCA	HIST1H2AC	47	546	11.61702128

Table 5: List of the rare mutations within the predicted head and neck squamous cell carcinoma driver genes. The mutations are filtered by minor allele frequency (MAF) in ExAC of <0.01.

Gene Name	Mutations	Number of patients share this mutation	ExAC_Freq	SIFT score	Polyphen2 score	MutationTaster score	MutationAssessor score	FATHMM score	VEST3 score	Unscaled/Raw CADD	PHRED-scaled CADD	Type	Prediction
TGM3	rs114998364	1	0.0027	0.844	0.001	1	0.345	-0.16	0.066	0.429	6.863	heterozygous	tolerated
TGM3	rs200294064	1	0.6×10^{-4}	0.063	0.936	1	1.965	1.52	0.618	4.825	24.8	heterozygous	deleterious
TGM3	rs539884508	1	0.0004	0.595	0.002	1	0.55	-0.72	0.046	-1.38	0.004	heterozygous	tolerated
OSGIN1	rs138964906	1	0.0007	0.83	0.038	0.997	0.9	1.34	0.05	1.214	11.82	heterozygous	tolerated
TNKS1BP1	rs747045370	1	0	0.269	0.042	1	0.345	1.61	0.106	0.415	6.743	heterozygous	tolerated
TNKS1BP1	rs115694714	3	0.0030	0.007	0.993	0.626	0.695	0.98	0.463	4.699	24.6	heterozygous	deleterious
TNKS1BP1	rs79359831	2	0.0032	0.121	0.017	1	1.61	0.97	0.189	1.967	16	heterozygous	deleterious
TNKS1BP1	rs748159115	1	0	0.535	0.025	1	0.41	1.45	0.136	0.7	8.826	heterozygous	tolerated
CCNA1	rs113565588	3	0.0002	0.306	0.001	1	0	2.39	0.151	-0.001	2.574	heterozygous	tolerated
TAPBP	rs145837211	3	0.0052	0	0.002	1	.	1.32	0.101	3.795	23.4	heterozygous	cannot assess

Table 6: List of the rare mutations within the predicted colorectal driver genes. The mutations are filtered by minor allele frequency (MAF) in ExAC of <0.01.

Gene name	Mutation	Number of patients share this mutation	ExAC_Freq	PolyPhen2 score	SIFT score	MutationAssessor score	MutationTaster score	FATHMM score	VEST3 score	Unscaled/Raw CADD	PHRED-scaled CADD	Type	Prediction
FES	rs761486582	2	0	0.988	0.003	1.205	1	-1.67	0.532	3.615040	25.5	heterozygous	deleterious
OBSCN	rs377742814	2	0.3×10^{-4}	0.989	0.03	2.61	1	-0.27	0.588	2.928777	23.5	heterozygous	deleterious
OBSCN	rs201725133	2	0.8×10^{-4}	.	.	.	1	.	.	6.609900	36	heterozygous	cannot assess
OBSCN	rs775164465	2	0	0.001	0.525	0.02	1	0.62	0.131	-0.369758	0.224	heterozygous	deleterious
OBSCN	rs372037309	2	0.5×10^{-4}	0.737	0	1.78	1	1.07	0.388	3.478409	25	heterozygous	deleterious
OBSCN	rs373638525	2	0.00012	0.989	0.022	0.885	0.867	-0.21	0.317	3.502569	25.1	heterozygous	deleterious
OBSCN	rs770745639	2	0	0.023	0.026	0.95	0.999	-0.01	0.062	3.252189	24.3	heterozygous	deleterious
OBSCN	rs776318810	2	0	0.029	0.218	1.505	1	-0.23	0.186	0.434124	8.744	heterozygous	tolerated
OBSCN	rs749249863	2	0	0.877	0.215	0.655	1	-0.28	0.196	2.196906	21.3	heterozygous	tolerated
OBSCN	rs188024384	2	0.0015	0.105	0.139	0.69	1	-0.21	0.258	0.680169	10.93	heterozygous	deleterious
OBSCN	rs756875131	2	0	0.904	0.085	2.385	1	-0.35	0.177	-0.091695	1.480	heterozygous	deleterious
OBSCN	rs201854668	2	0.00124	0.999	0.088	2.045	1	-0.2	0.416	7.344976	38	heterozygous	deleterious
OBSCN	rs188531451	2	0.8×10^{-4}	0.974	0.051	2.465	1	0.29	0.31	3.299496	24.4	heterozygous	deleterious
OBSCN	rs559123841	2	0.8×10^{-4}	.	0.687	.	1	3.72	0.025	-0.020337	2.326	heterozygous	cannot assess
OBSCN	rs62621832	2	0.00709	1	0	3.14	1	2.1	0.664	3.530120	25.2	homozygous	deleterious
OBSCN	rs199501426	2	0.00022	0.156	0.071	2.305	1	-0.3	0.499	2.536169	22.7	heterozygous	deleterious

Table 7: List of overlapping predicted driver genes with the current COSMIC confirmed cancer driver genes.

Gene Name
MAPK1
STIL
ETNK1
TAL1
RUNX1T1
PIM1
FES
DAXX

Table 8: Prediction performances of applying OPA2Vec on the combined ontology using different embedding sizes and minimum counts evaluated by AUC. AUC is reported on a 20% test set randomly chosen from the positives and negatives.

Parameters setting	F-score	AUC
embedding size = 50, window size = 5, min-count = 0	89.06%	91.13%
embedding size = 100, window size = 5, min-count = 0	91.15%	93.87%
embedding size = 200, window size = 5, min-count = 0	90.90%	91.49%
embedding size = 300, window size = 5, min-count = 0	91.33%	92.33%
embedding size = 50, window size = 5, min-count = 5	88.42%	90.81%
**embedding size = 100, window size = 5, min-count = 5**	**92.57%**	**94.28%**
embedding size = 200, window size = 5, min-count = 5	91.11%	91.37%
embedding size = 300, window size = 5, min-count = 5	91.68%	92.74%
